# Egg albumin enhances kappa (κ)-carrageenan hydrogels for tunable physicochemical, mechanical, and biological performance

**DOI:** 10.1038/s41598-025-25208-9

**Published:** 2025-11-21

**Authors:** K. Likhith, Tarun Mateti, Goutam Thakur

**Affiliations:** 1 Department of Biomedical Engineering, Manipal Institute of Technology, Manipal Academy of Higher Education, Manipal, Udupi, 576104 Karnataka India; 2 Department of Mechanical Engineering & Materials Science, Yale School of Engineering & Applied Science, Yale University, 17 Hillhouse Avenue, New Haven, Connecticut, 06520 USA

**Keywords:** Drug delivery, Controlled drug release, Tissue engineering, Wound healing, Bioactivity, Biodegradable, Biomaterial, Biocompatibility, Biological techniques, Materials science

## Abstract

Hydrogels are versatile polymeric materials valued for their crosslinked structures, which mimic the extracellular matrix. Their tunable size, shape, and pore architecture make them attractive for diverse biomedical applications. In this study, we developed a novel biohybrid hydrogel composed of κ-carrageenan and egg albumin. Egg albumin, extracted from fresh egg whites (*Gallus gallus domesticus*), was quantified using the Bradford assay. Protein molecular masses were characterized by sodium dodecyl sulfate–polyacrylamide gel electrophoresis (SDS–PAGE) and confirmed via matrix-assisted laser desorption/ionization time-of-flight mass spectrometry (MALDI–TOF–MS). Hydrogel scaffolds were fabricated through ionotropic gelation, forming an interpenetrating polymer network of κ-carrageenan and egg albumin, with sodium chloride (NaCl) and potassium chloride (KCl) from phosphate-buffered saline (PBS) serving as crosslinking agents. Fourier-transform infrared spectroscopy (FTIR) was used to assess polymer crosslinking, and scaffold stability was evaluated through swelling behavior, degradation rate, and mechanical strength. Surface morphology analysis revealed a dense, folded network in pure κ-carrageenan hydrogels, whereas egg albumin incorporation yielded smoother surfaces with protein aggregates—features that increase surface heterogeneity and may promote cell adhesion. 3-(4,5-dimethylthiazol-2-yl)-2,5-diphenyltetrazolium bromide (MTT) assays with NIH 3T3 (mouse embryonic fibroblast) cells demonstrated significantly higher cell proliferation on egg albumin-blended hydrogels compared with pure κ-carrageenan on days 1, 4, and 7. These results indicate that egg albumin–copolymerized hydrogels are promising candidates for soft tissue engineering and drug delivery applications.

## Introduction

Tissue engineering aims to restore or replace damaged tissues through the integration of cells, scaffold materials, and bioactive mediators^1^. Scaffolds act as structural frameworks that enable cell adhesion, proliferation, and spatial organization, while bioactive molecules such as growth factors and cytokines regulate cellular behavior and direct tissue formation^2,3^. Such systems are particularly valued for their ability to mimic the extracellular matrix (ECM), promoting biocompatibility, regenerative potential, and seamless integration with host tissues^4,5^. Among these, hydrogels—porous scaffolds with > 90% water content—closely resemble natural ECM, supporting cell attachment, regulating molecular interactions, maintaining mechanical integrity, and undergoing controlled biodegradation^6–8^. Scaffold performance depends on material composition, surface chemistry, molecular weight, solubility, and degradation mechanisms^9–11^.

Polymeric materials provide structural support for cell attachment and tissue development. Classified as natural or synthetic, they are favored for their abundance, ease of fabrication, tunable properties, biodegradability, and biocompatibility^12^. Synthetic polymers such as polycaprolactone (PCL), polyglycolic acid (PGA), polylactic acid (PLA), polyvinyl alcohol (PVA), and polyurethane (PU) offer excellent physicochemical and mechanical properties but may cause cytotoxicity, exhibit biological inertness, and lack intrinsic therapeutic functions^13^. Their limited cell–material interactions often require surface modification or blending with natural biomaterials to improve biocompatibility^14^. Naturally derived polymers from plant, animal, and microbial sources possess functional groups that enable tuning through physical interactions and chemical crosslinking^15^. These natural polymers are biodegradable, biocompatible, and low in antigenicity, with some also exhibiting antimicrobial and anti-inflammatory activity that supports tissue repair and regeneration^16^.

Carrageenan is a notable natural polymer with versatile chemical structures that can be tailored for specific applications and enzymatically degraded into nontoxic byproducts^17,18^. Its high fluid absorption, biocompatibility, and biodegradability make it an attractive scaffold material. κ-Carrageenan (KC) (classified by its single sulfate group per disaccharide unit) forms compact gels via ionic interactions and hydrogen bonding with cations such as K⁺, Ca²⁺, and Na⁺. Gelation upon cooling in the presence of K⁺ produces a structurally stable hydrogel, though stability decreases at elevated temperatures. These gelling properties, combined with biocompatibility and versatility, highlight KC’s potential in biomedical scaffold development^6^.

Egg albumin (EA) is a versatile biomaterial with high bioactivity, biocompatibility, ease of handling, broad availability, and low processing costs. EA exhibits foaming, gelling, and emulsifying properties that allow fabrication into sponges, hydrogels, films, fibers, nanogels, and particles. However, its mechanical strength is limited by its composition—primarily water (84–89%) and proteins (10–11%), with minor amounts of carbohydrates, lipids, and minerals^19^. Major proteins include ovalbumin (54%), ovotransferrin (12–13%), ovomucoid (11%), ovoglobulins (2%), ovomucin (1.5–3%), and lysozyme (3.5%), alongside trace proteins such as ovastatin, ovoflavoproteins, and avidin. EA also contains enzymes (e.g., lysozyme, phosphatase, catalase, glycosidases), vitamins (biotin, niacin, riboflavin), minerals (S, K, Na, Cl, with trace Ca, P, Mg), minimal lipids (0.03% w/w; oleic, palmitic, arachidonic, linoleic, stearic acids), and carbohydrates in the form of glucose, oligosaccharides, and glycoproteins^20^.

Recently, protein–polysaccharide hybrid hydrogels have gained increasing attention for biomedical applications because the complementary characteristics of both components can be exploited to enhance scaffold performance. For instance, κ-carrageenan has been blended with proteins such as gelatin^21^, collagen^22^, phycocyanin^23^, whey protein^24^, fibrinogen hydrolysate^25^, silk fibroin^26^, casein^27^, mussel adhesive protein (Pvfp5β)^28^, pectin^29^, scallop protein hydrolysates^30^, soy protein^31^, and β-lactoglobulin amyloid fibrils^32^ to improve mechanical stability, bioactivity, or biodegradability. Most of these systems rely on single purified proteins that contribute specific structural or biochemical features—for example, the triple-helix motifs of gelatin or the β-sheet domains of silk fibroin that enhance mechanical strength and elasticity. However, such proteins often require chemical crosslinkers, are expensive to isolate, or lack multifunctional biochemical activity. In contrast, egg albumin represents a complex natural protein mixture, which together provide a diverse array of reactive and bioactive groups. This intrinsic heterogeneity enables non-covalent interactions with κ-carrageenan without the need for external crosslinkers, resulting in tunable hydrogels that are cost-effective, easy to fabricate, and enriched with cell-recognition sequences and enzymatically degradable motifs.

While prior studies have independently explored KC or EA, their combination as a biohybrid scaffold material has not been systematically investigated. Our work introduces EA as a new, low-cost, and bioactive copolymer in KC-based hydrogels and comprehensively evaluates how its incorporation influences physicochemical, mechanical, and biological properties. This establishes a foundation for using EA to enhance scaffold bioactivity, which has not been addressed in earlier studies. The EA–KC combination is expected to enhance the functional performance of KC hydrogels, improving their suitability for biomedical scaffolding. We assessed the impact of EA incorporation on the physicochemical properties of the hydrogels and conducted comprehensive characterization, including surface morphology, chemical interactions, mechanical strength, and water retention capacity—key parameters in determining scaffold performance for tissue engineering.

## Materials and methods

### Materials

κ-Carrageenan (Total carbohydrate 90 wt%; 6-anhydro-D-galactose content, 36.5%; sulfate ester content, 13.1%; viscosity 8–12 cP, 0.3% H₂O, 25 °C; Mₙ 4.20 × 10⁵ Da; SNAP Natural and Alginate Products Pvt. Ltd., Chennai, India) was used in this study. Fresh egg whites (*Gallus gallus domesticus*) were sourced locally and processed as described in Sect. 2.2. Dialysis was performed using SnakeSkin™ Dialysis Tubing, 3.5 kDa MWCO, regenerated cellulose, nominal dry thickness 22–30 μm, sulfur content 0.1–0.15%, heavy metals trace (Thermo Fisher Scientific, Catalog No. 68035). Trypsin–EDTA (0.25%) with phenol red was purchased from Gibco™ (Thermo Fisher Scientific, Catalog No. 25200056). Sodium chloride (NaCl; CAS No. 7647-14-5), potassium chloride (KCl; CAS No. 7447−40-7), potassium dihydrogen phosphate (KH₂PO₄; CAS No. 7778-77-0), and disodium hydrogen phosphate (Na₂HPO₄; CAS No. 7782-85-6) were obtained from Merck (India). All experiments were conducted using deionized, double-distilled water.

### Egg albumin isolation and characterization

Fresh hen eggs were sourced from a local grocery store in Manipal. Egg surfaces were sanitized with 70% ethanol to maintain sterility and minimize microbial contamination. A ~ 1 cm opening was created by carefully cracking each egg, and the egg white was gently separated into a beaker. It was diluted 1:1 with double-distilled water to reduce viscosity and facilitate downstream processing, and then filtered through gauze to remove particulates and large debris. The filtrate was stirred magnetically in an ice bath for 1 h to ensure homogeneity while preserving protein integrity by preventing heat-induced denaturation. It was then centrifuged at 10,000 rpm for 20 min at 4 °C to remove insoluble matter. The supernatant was collected and dialyzed overnight against distilled water at 4 °C using a 3.5 kDa MWCO regenerated cellulose membrane (Thermo Fisher, USA), with four water changes, to remove salts and other low-molecular-weight contaminants while retaining proteins. The dialyzed solution was centrifuged again at 15,000 rpm for 40 min at 4 °C to remove residual impurities and aggregates. The final protein solution was lyophilized for 48 h to obtain a stable dry powder and stored at 4 °C until use^33^.

SDS–PAGE was conducted using a 12% resolving gel, following the Laemmli method. Gels were stained with Coomassie Brilliant Blue R-250 and destained in a solution of 50% (v/v) methanol and 10% (v/v) glacial acetic acid. Protein molecular weights were estimated by comparing band migration to Prestained Protein Molecular Weight Markers (Thermo Scientific, USA; 20–120 kDa range, six bands). Protein concentrations were quantified using the Bradford assay, with bovine serum albumin (BSA) as the standard^34^.

Protein molecular masses were determined using MALDI–TOF mass spectrometry on a Bruker ultrafleXtreme instrument (Germany). Samples were digested overnight at 37 °C with trypsin. Following digestion, 1.0 µL of the peptide solution was mixed with 1.0 µL of a saturated matrix solution containing recrystallized α-cyano-4-hydroxycinnamic acid in 50% (v/v) acetonitrile and 0.1% (v/v) trifluoroacetic acid (TFA). A 0.3 µL aliquot of this mixture was spotted onto a MALDI target plate and air-dried at room temperature (28 °C). Spectra were acquired over a mass range of 5,000–55,000 Da, and calibration was performed using the Bruker Peptide Calibration Standard II^35^.

###  Fabrication of KC–EA hydrogels

A 3% (w/v) κ-carrageenan (KC) solution was prepared in phosphate-buffered saline (PBS; pH 7.4) at 60 °C under continuous magnetic stirring. The concentration of 3% KC was chosen based on optimization trials, as higher concentrations (> 3%) led to premature gelation within the beaker during preparation, whereas lower concentrations (≤ 2%) produced weak and unstable gels. The solution was then cooled to 40 °C. Lyophilized EA powder was added in varying amounts (1% and 2% w/v relative to KC) and mixed thoroughly to ensure homogeneous dispersion (Table 1). Care was taken to maintain this lower incorporation temperature, as higher temperatures can lead to denaturation of EA proteins and loss of their biochemical functionality. These concentrations were selected to investigate the effect of increasing protein incorporation, since higher EA loading yielded excessively soft hydrogels with poor structural stability. The resulting mixtures were cast into molds (35 mm × 15 mm) and allowed to cool to 28 °C for 30 min to complete gelation.

**Table 1:** Composition of κ-carrageenan (KC)–egg albumin (EA) hydrogels. Three formulations were prepared with constant KC content (3 g) and varying EA concentrations to achieve KC: EA ratios of 3:0 (hydrogel A), 3:1 (hydrogel B), and 3:2 (hydrogel C).

Hydrogel	Ratio of KC and EA	Quantity of KC (g)	Quantity of EA (g)
**A**	3:0	3	0
**B**	3:1	3	1
**C**	3:2	3	2

### Swelling behavior

The swelling behavior of the hydrogels was evaluated by immersing pre-weighed dry samples in double-distilled water at 28 °C under static conditions. At designated intervals, samples were removed, surface water was gently blotted, and the swollen hydrogels were weighed until equilibrium swelling was reached^36^. Saturation was determined visually, when no further observable change in hydrogel volume or weight occurred across consecutive measurements. The swelling ratio was calculated using Eq. (1):

1 $$\:\%\:Swelling\:=\:\frac{{w}_{t}\:-\:{w}_{0}}{{w}_{0}}\:\times\:\:100$$

where wₜ is the weight of the hydrogel at time t and w₀ is the initial dry weight of the hydrogel.

### Functional group analysis

Fourier transform infrared spectroscopy (FTIR) was used to identify functional groups and molecular interactions within the hydrogel. Oven-dried samples (3 mg) were analyzed in transmittance mode using a Shimadzu IR Spirit-8300 spectrometer (Japan). Spectra were collected over the range of 4000–400 cm⁻¹ with 32 scans at a resolution of 4 cm⁻¹^37^. All spectra were baseline-corrected, smoothed, and normalized prior to analysis to ensure accurate comparison of peak positions and relative intensities.

### Surface morphology analysis

Hydrogel surface morphology was examined using a scanning electron microscope (SEM; Carl Zeiss EVO MA 18, Germany) equipped with an Oxford EDS X-act detector. A 1 × 1 cm hydrogel sample was thoroughly dried, sputter-coated with gold for 1 min, and imaged at various magnifications under vacuum at an accelerating voltage of 10 kV^38^, focusing on surface morphology and topographical features.

### Thermogravimetric analysis

Thermal stability of the hydrogels was evaluated using a thermal analyzer (STA 7200, Hitachi High-Tech Science, Japan). Finely ground samples (0.5–5 mg) were placed in an alumina pan coupled to a microbalance for accurate mass measurement. Thermogravimetric analysis (TGA) was conducted from 30 to 800 °C at a heating rate of 10 °C/min under a continuous nitrogen atmosphere^39^.

### Strength analysis

Mechanical properties of the hydrogels were evaluated using a texture analyzer (EZ-SX, Shimadzu, Japan). Cylindrical samples (35 mm diameter × 15 mm height) were tested at 28 °C in the hydrated state. Compression was applied at a loading rate of 1 mm/min using a 500 N load cell. Stress–strain curves were recorded and used to calculate total energy, Young’s modulus, and compressive strength^40^. All measurements were performed in triplicate (*n* = 3).

### Physiological stability

Hydrogel degradation was assessed by immersing pre-weighed samples in phosphate-buffered saline (PBS; pH 7.4) at 37 °C under continuous agitation in a shaking incubator. At predetermined intervals of 24 h over one month, samples were removed, gently blotted with filter paper, and reweighed^6^. PBS was not refreshed during the study, as maintaining a constant medium was intended to mimic in vivo conditions where degradation products accumulate locally. The percentage weight loss was calculated using Eq. (2):

2 $$\:\%\:Degradation\:=\:\frac{{w}_{s}\:-\:{w}_{i}}{{w}_{s}}\:\times\:\:100$$

where wₛ is the initial weight of the hydrogel before incubation and w_i_ is the weight after incubation in PBS.

### In vitro cytotoxic assessments

Cytotoxicity was evaluated using the MTT assay. NIH 3T3 cells were cultured on UV-sterilized hydrogels for 7 days following standard protocol. Cells were seeded at a density of 3.125 × 10³ cells/cm² in Dulbecco’s Modified Eagle’s Medium (DMEM) supplemented with 10% fetal bovine serum and 1% penicillin–streptomycin. Tissue culture polystyrene (TCP) wells seeded with untreated cells served as the control. MTT solution (0.5 mg/mL) was added to the scaffolds and incubated for 4 h at 37 °C in 5% CO₂. The resulting formazan crystals were dissolved in dimethyl sulfoxide (DMSO), and absorbance was measured using a multimode microplate reader (Ensight HH34000000, PerkinElmer, USA).

A live/dead viability assay (Thermo Fisher, USA) was also performed, with TCP-seeded cells serving as the control to assess cell viability. Sterilized scaffolds were seeded with 3T3 cells and incubated for 1 h at 37 °C in a staining solution containing calcein-AM and ethidium homodimer-1. Fluorescence images were captured using an inverted fluorescence microscope (Eclipse Ts2-FL, Nikon, Japan), and cell viability was quantified as the percentage of live cells relative to the total cell population^40^.

### Statistical analysis

All experiments were carried out in triplicate, and the results were expressed as arithmetic mean ± standard deviation (SD). Statistical analyses were performed using GraphPad Prism 7.0 software (GraphPad Software, La Jolla, CA, USA). One-way analysis of variance (ANOVA) followed by Tukey’s post-hoc test was applied to evaluate differences between groups, and a p-value < 0.05 was considered statistically significant.

## Results and discussion

### Isolation of egg albumin and characterization

**Fig. 1:**
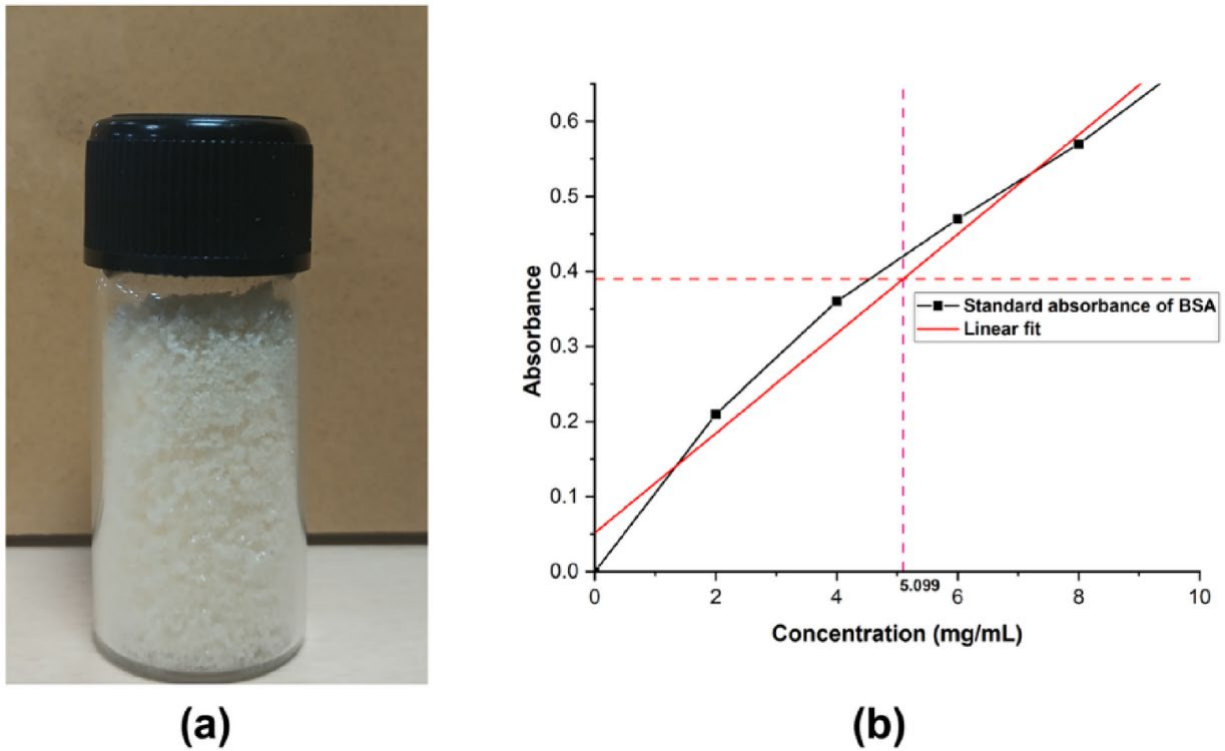
(**a**) Lyophilized egg albumin powder obtained after centrifugation, dialysis, and freeze-drying of egg white proteins. (**b**) Determination of total protein concentration (~ 5.10 mg/mL) by Bradford assay using bovine serum albumin (BSA) as the standard.

EA was isolated by centrifugation of egg white at 4 °C, followed by overnight dialysis of the supernatant. The resulting protein mixture was lyophilized (Fig. 1*(a)*) and stored at 4 °C. The total protein concentration was approximately 5.10 mg/mL (Fig. 1*(b)*), confirming the protein-rich nature of hen egg white.

**Fig. 2:**
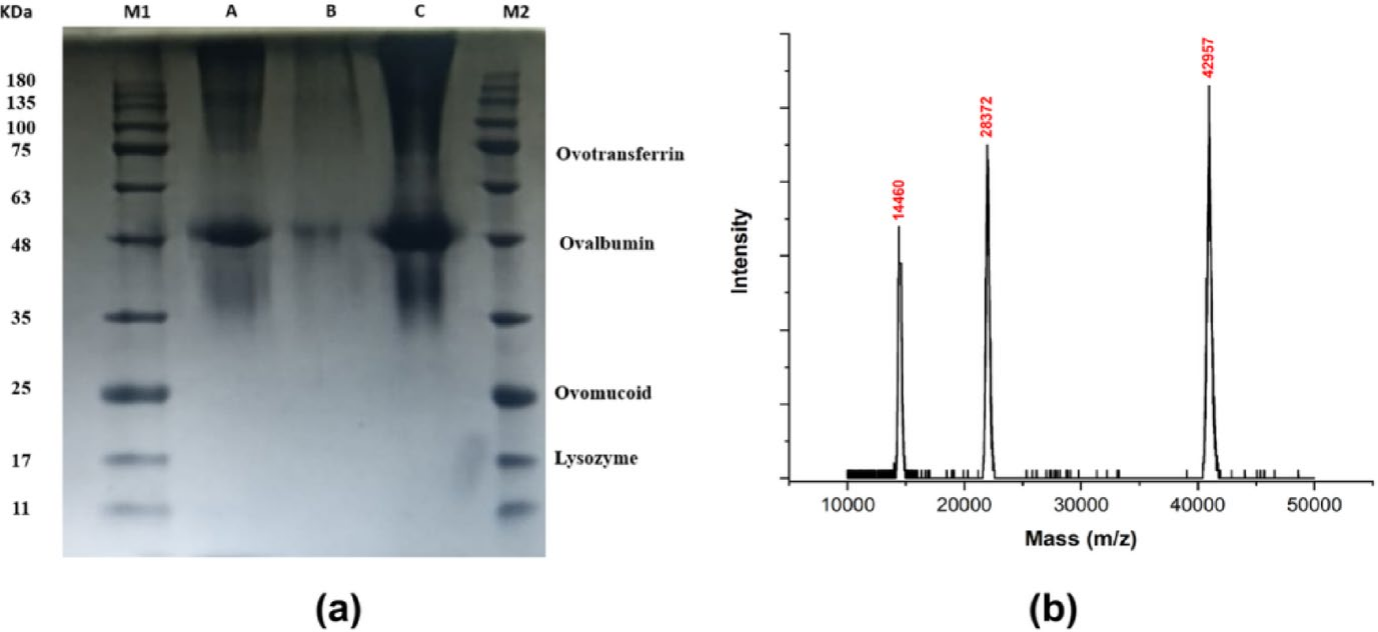
(**a**) SDS–PAGE analysis of egg albumin proteins on a 12% acrylamide gel. Lane 1: molecular weight marker; Lane 2: 10 µL sample; Lane 3: 5 µL sample; Lane 4: 15 µL sample; Lane 5: molecular weight marker. Prominent bands correspond to ovotransferrin (~ 77 kDa) and ovalbumin (~ 43–46 kDa). Bands for ovomucoid (~ 28 kDa) and lysozyme (~ 14 kDa) were faint or undetectable. (**b**) MALDI–TOF–MS spectrum of lyophilized egg albumin confirming the presence of major proteins, with peaks observed at characteristic molecular masses.

SDS–PAGE analysis (Fig. 2*(a)*) revealed distinct bands corresponding to the major egg white proteins^33^. Ovalbumin, the predominant protein (~ 54% of total protein), was identified by its electrophoretic mobility relative to molecular weight markers and appeared as the most intense band^41^. Another major band corresponded to ovotransferrin (conalbumin), which accounts for ~ 12% of egg white proteins^20^. Bands for ovomucoid (~ 11%) and lysozyme (~ 3.5%) were not detected. Their absence can be attributed to multiple factors: (i) their relatively low concentrations, which may fall below the microgram detection threshold of Coomassie staining, (ii) the small molecular size of lysozyme (~ 14 kDa), which can result in faint bands or migration beyond the gel under certain electrophoretic conditions, and (iii) possible aggregation or partial degradation during sample preparation, leading to poor separation or retention in wells. In contrast, MALDI–TOF–MS (Fig. 2(b)) is capable of detecting proteins and peptide fragments at nanogram to picogram levels, providing higher sensitivity and confirming the presence of both ovomucoid and lysozyme.

**Fig. 3:**
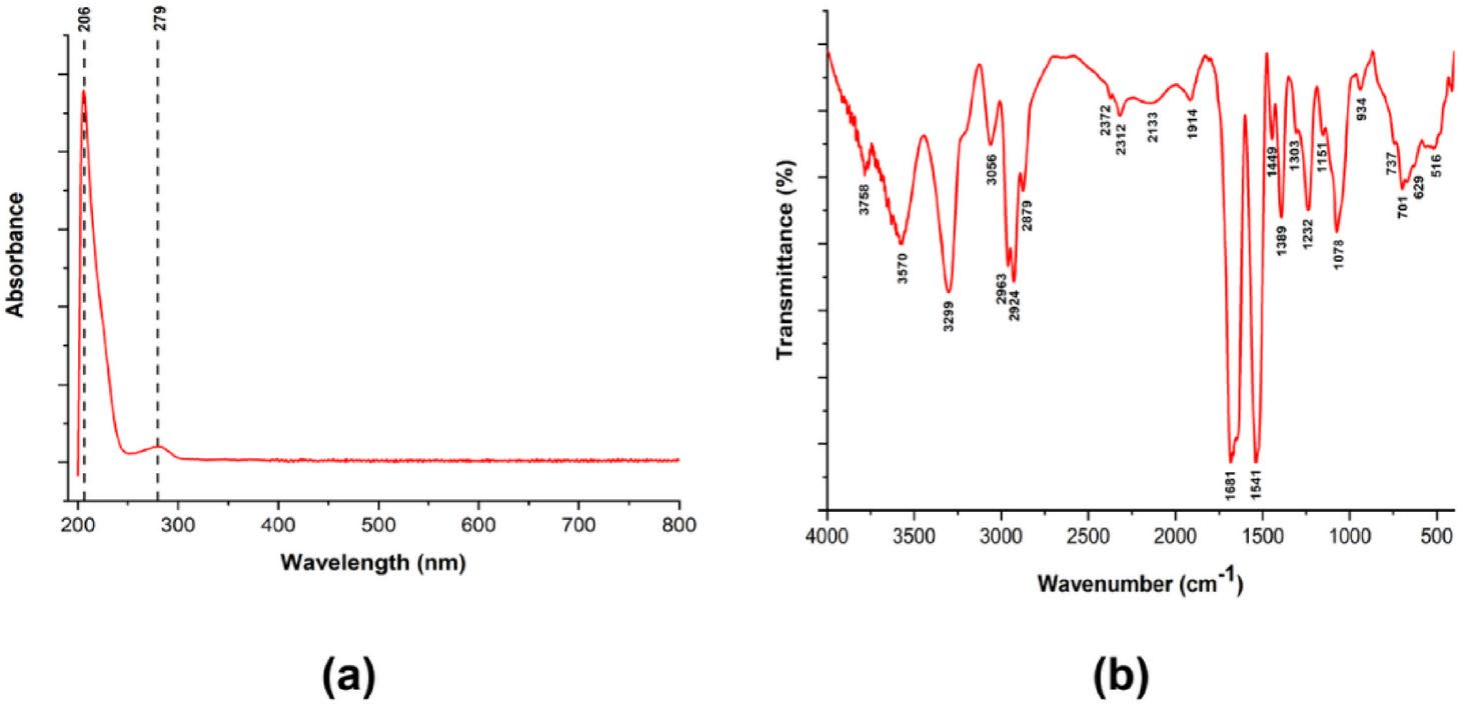
(**a**) UV–Vis spectrum of lyophilized egg albumin showing a characteristic absorbance peak at ~ 279 nm due to aromatic amino acids (phenylalanine, tryptophan, tyrosine) and a secondary peak at 200–210 nm attributed to peptide bonds. (**b**) FTIR spectrum of lyophilized egg albumin displaying characteristic absorption peaks of protein secondary structures, including the amide I band (1681 cm⁻¹, C = O stretching) and the amide II band (1541 cm⁻¹, C–N stretching and N–H bending).

The UV–Vis spectrum (Fig. 3*(a)*) of lyophilized EA showed a characteristic absorbance peak at ~ 279 nm, corresponding to aromatic amino acids (phenylalanine, tryptophan, and tyrosine). A secondary peak between 200 and 210 nm was attributed to peptide bonds^42^. The IR spectrum of EA (Fig. 3*(b)*) displayed characteristic absorption peaks associated with protein secondary structures. A prominent peak at 1681 cm⁻¹ corresponded to C = O stretching vibrations of the amide I band, a key feature in protein secondary structure analysis. Another peak at 1541 cm⁻¹ was assigned to C–N stretching coupled with N–H bending vibrations of the amide II band^43^.

### Physio-morphological properties of KC–EA hydrogels

**Fig. 4:**
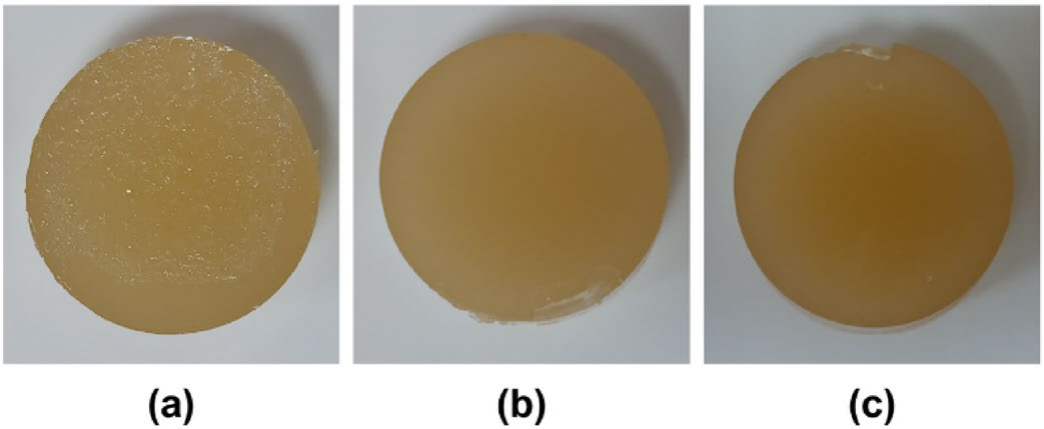
Photographs of hydrogels prepared by ionotropic gelation: (**a**) κ-carrageenan hydrogel, (**b**) κ-carrageenan hydrogel containing 1% (w/v) egg albumin, and (**c**) κ-carrageenan hydrogel containing 2% (w/v) egg albumin. The images illustrate the formation of stable, free-standing hydrogels with increasing protein incorporation.

KC–EA hydrogels (Fig. 4) were synthesized using the ionotropic gelation method. Gelation occurs through a two-step mechanism regulated by temperature and cations. At high temperatures (> 70 °C), KC exists in a random coil conformation due to electrostatic repulsion between adjacent chains. Upon cooling, the chains adopt a helical structure, and in the presence of cations, these helices aggregate into dimers, forming a stable hydrogel network^6^.

The choice of PBS as the solvent was critical for stable gel formation. KC is a cation-selective biopolymer that readily gels in the presence of large univalent cations such as K⁺, while responding weakly to smaller cations such as Na⁺. This behavior reflects ion-specific hydration: K⁺ (negative Jones–Dole B coefficient) disrupts water structure and promotes negative hydration, whereas Na⁺ (positive coefficient) enhances structure ordering and positive hydration^44^. PBS, which contains both Na⁺ and K⁺, therefore supports KC gelation without requiring chemical crosslinkers. Moreover, EA proteins are soluble in neutral, dilute salt solutions, with NaCl further improving solubility. Thus, PBS serves as an effective medium for dissolving EA and incorporating it uniformly into the KC hydrogel matrix.

#### Swelling analysis

Swelling is a key property of hydrogels, as it governs water absorption, exudate uptake, and the diffusion of therapeutic agents through the polymer network. This behavior depends largely on the abundance of hydrophilic functional groups and the structural organization of the hydrogel matrix^45^. The synthesized KC–EA hydrogels, being both hydrophilic and flexible, readily absorbed water and exhibited substantial swelling.

**Fig. 5:**
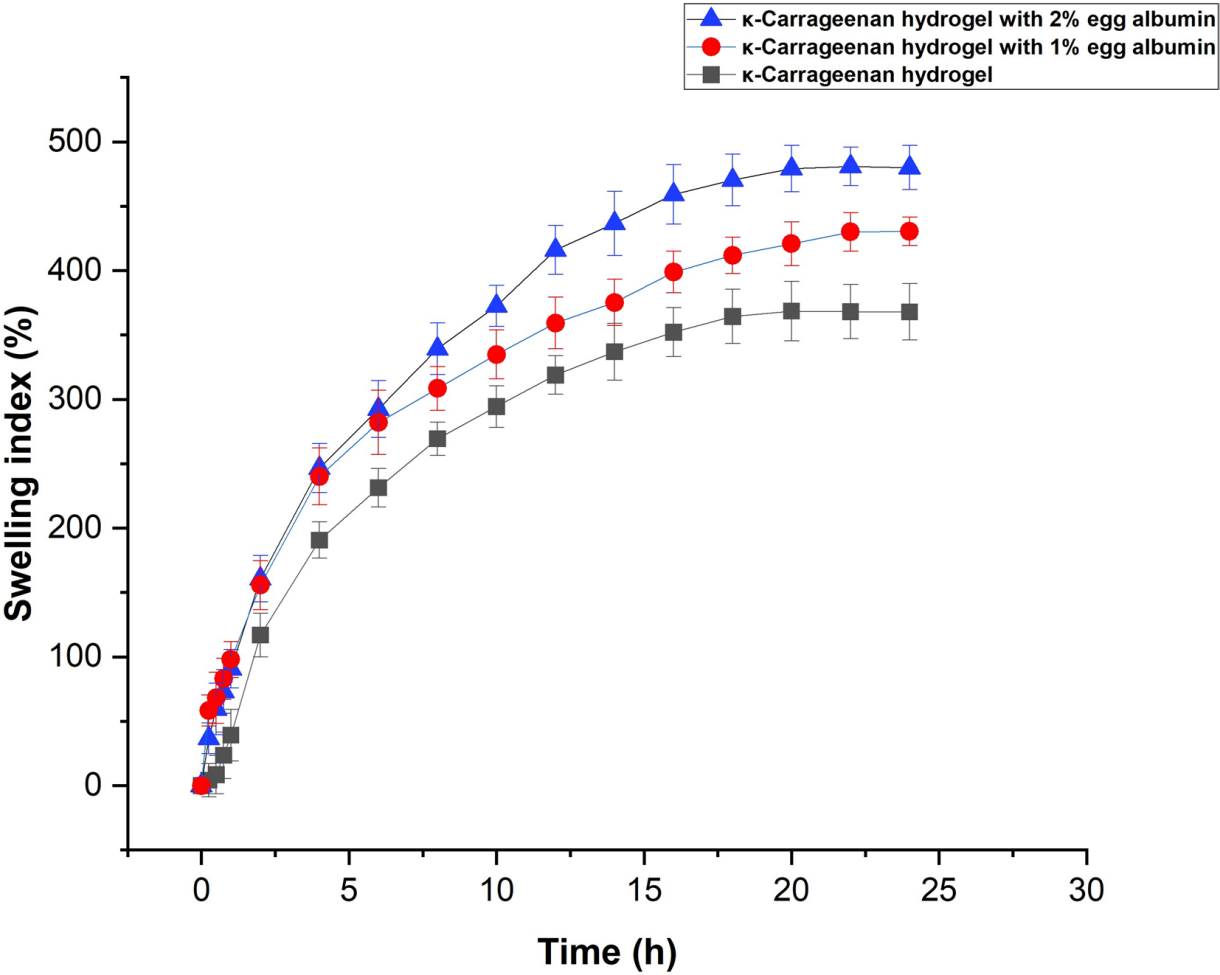
Swelling behavior of κ-carrageenan (KC) and KC–egg albumin (EA) hydrogels in double-distilled water at 28 °C. Pure KC hydrogels showed a lower swelling index compared with EA-blended hydrogels. The addition of 1% EA increased swelling capacity, while 2% EA initially enhanced water uptake but led to reduced stability and progressive degradation during prolonged soaking. Data are presented as mean ± SD (*n* = 3).

Figure 5 presents the swelling behavior of KC and KC–EA hydrogels. Pure KC hydrogels showed a lower swelling index compared with EA-blended hydrogels, reflecting the additional hydrophilic groups and porous structure contributed by proteins. The swelling rate increased rapidly with time and reached equilibrium at ~ 18 h. Between the protein-loaded systems, the hydrogel containing 2% EA exhibited higher swelling than that with 1% EA. The increased swelling of the 2% EA hydrogel was attributed to its higher protein content, which increased water uptake and gel extensibility but also weakened intermolecular interactions within the polymer network. As a result, the 2% EA hydrogel showed progressive structural degradation during prolonged soaking^46^.

#### Functional group analysis

**Fig. 6:**
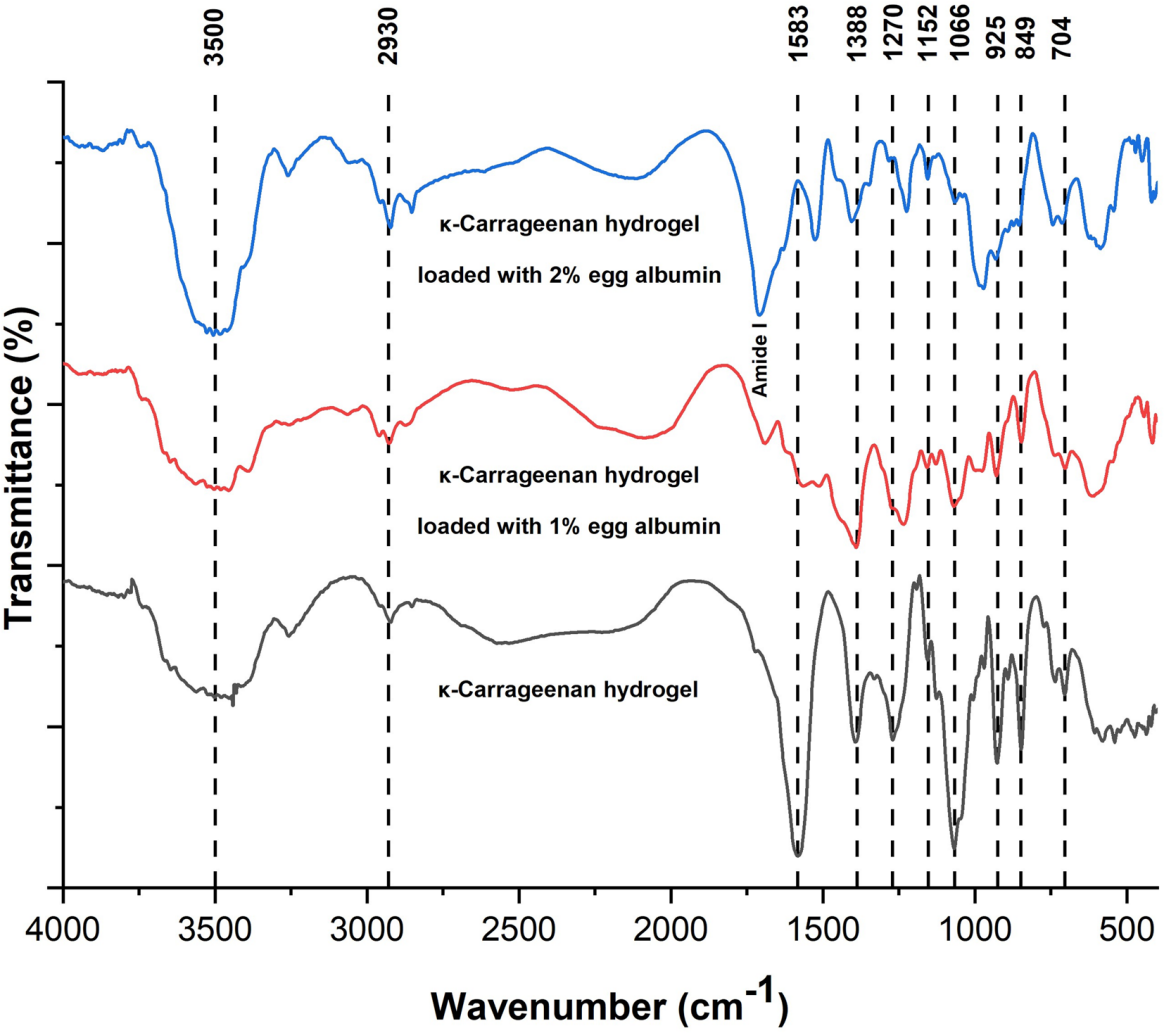
FTIR spectra of κ-carrageenan (KC) hydrogel and KC–egg albumin (EA) hydrogels containing 1% and 2% (w/v) egg albumin. KC exhibited characteristic peaks at 3400–3600 cm⁻¹ (O–H stretching), 2930 cm⁻¹ (C–H stretching), 1583 cm⁻¹ (C = O stretching), 1270 cm⁻¹ (S = O stretching of sulfate esters), and 925 and 849 cm⁻¹ (C–O–C and C4–O–S stretching of 3,6-anhydrogalactose). Upon blending with EA, the amide I (1700–1600 cm⁻¹) and amide II (1541 cm⁻¹) bands intensified, while the characteristic KC peaks weakened, indicating hydrogen bonding and electrostatic interactions between KC and EA.

FTIR spectroscopy (4000–400 cm⁻¹) was used to identify functional groups and possible interactions in the hydrogels (Fig. 6). The spectrum of KC exhibited a broad band at 3400–3600 cm⁻¹, corresponding to O–H stretching vibrations. Additional peaks were observed at 2930 cm⁻¹ (C–H stretching), 1583 cm⁻¹ (C = O stretching), 1388 cm⁻¹ (symmetric COO⁻ stretching), 1270 cm⁻¹ (S = O stretching of sulfate esters), 1152 cm⁻¹ (C–O–C stretching), 1066 cm⁻¹ (C–O stretching of glycosidic linkages), and 925 and 849 cm⁻¹ (C–O–C and C4–O–S stretching of 3,6-anhydrogalactose)^47^.

The reference spectrum of EA (Fig. 3*(b)*) showed characteristic amide peaks at 3299 cm⁻¹ (amide A), 1681 cm⁻¹ (amide I), and 1541 cm⁻¹ (amide II)^48^. Upon blending with KC, the broad O–H stretching band became more intense and expansive, indicating hydrogen bonding between hydroxyl groups of KC and EA. The amide I band (1700–1600 cm⁻¹) increased with EA concentration, confirming protein incorporation into the hydrogel network. Additionally, heating promoted conformational rearrangements in EA, with reductions in random coil and β-turn content and an increase in ordered α-helices and intermolecular β-sheets—structural changes commonly observed in heat-denatured proteins^49^.

The characteristic KC peaks at 925 and 849 cm⁻¹ became less intense upon blending, further demonstrating interactions between KC and EA^50^. These interactions are primarily driven by hydrogen bonding and electrostatic attraction between the negatively charged sulfate ester groups of KC and the positively charged domains of EA. Such interactions promote partial unfolding of EA, expose buried amino acids, and enhance water absorptivity, as supported by the swelling studies^51^.

#### Surface microstructure analysis

**Fig. 7:**
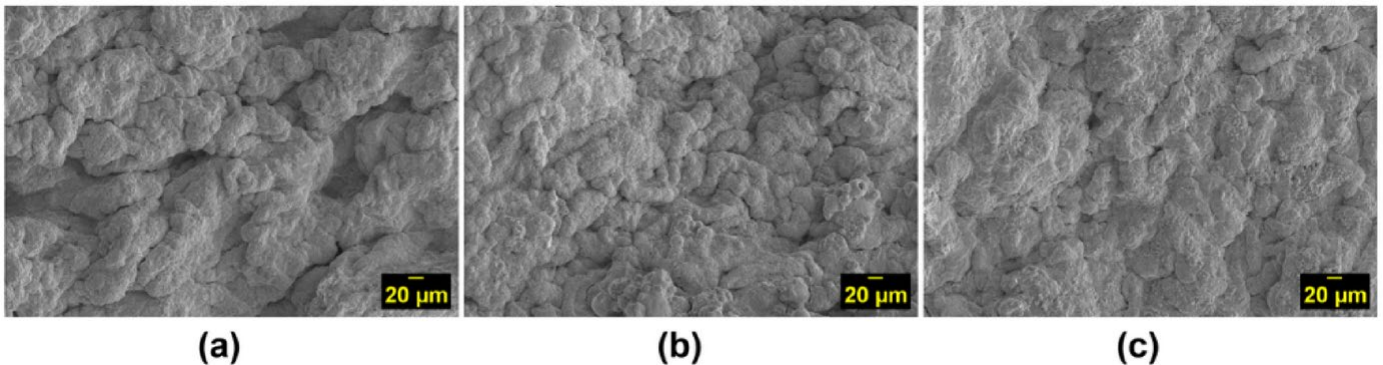
SEM micrographs of hydrogel surfaces at 500× magnification and 10 kV: (**a**) pure κ-carrageenan (KC) hydrogel, (**b**) KC hydrogel with 1% (w/v) egg albumin (EA), and (**c**) KC hydrogel with 2% (w/v) EA. Pure KC hydrogel displayed a dense and uniform network, while EA incorporation induced morphological changes, including surface roughness, folds, and spherical aggregates. These features became more pronounced at higher EA concentrations, indicating protein–polysaccharide interactions within the hydrogel matrix.

The surface morphology of KC–EA hydrogels was examined by SEM at 500× magnification and 10 kV (Fig. 7). Pure KC hydrogels exhibited a dense and uniform polymer network in the presence of K⁺, consistent with previous reports^52^. Incorporation of EA induced pronounced morphological changes, producing rougher surfaces with distorted regions (Fig. 7b–c). As EA concentration increased, the hydrogel surface became more irregular, with spherical aggregates attributed to ovalbumin, a major globular protein in EA. At higher EA content, these aggregates appeared larger and more organized, suggesting electrostatic interactions between negatively charged KC and positively charged EA proteins^38^.

The presence of folds and surface irregularities decreased with higher EA loading, indicating a loosening of the KC polymer network. This smoother but more heterogeneous surface morphology is consistent with swelling results, which showed that EA incorporation increased water uptake and hydrophilicity. Heat-induced rearrangements likely promoted protein–polysaccharide interactions, but these interactions weakened the overall network integrity rather than strengthening it, as reflected by reduced mechanical strength described later. Importantly, despite the softer structure, the increased surface heterogeneity and the presence of protein aggregates may enhance cell adhesion, underscoring the potential of KC–EA hydrogels for tissue engineering applications.

#### Thermal stability assessment

**Fig. 8:**
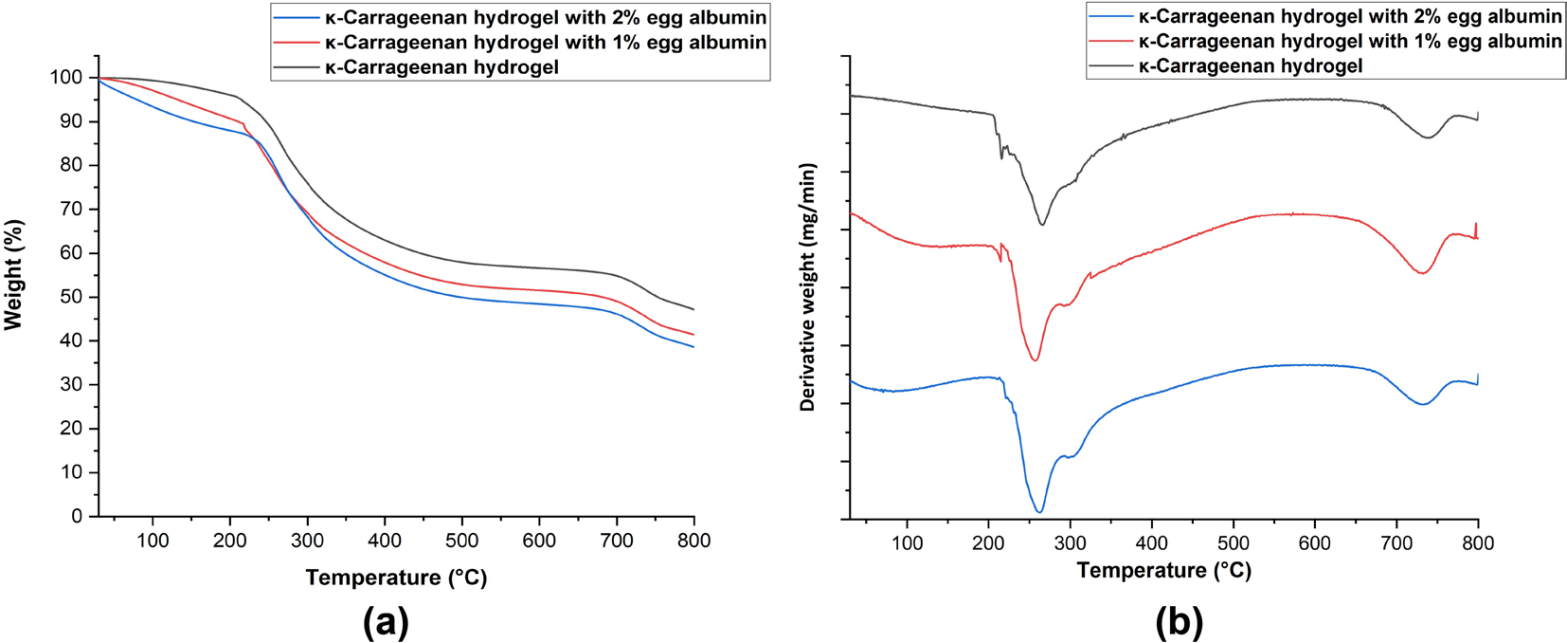
Thermal stability of KC–EA hydrogels evaluated by (**a**) thermogravimetric analysis (TGA) and (**b**) derivative thermogravimetry (DTG). TGA curves show two major weight-loss events: initial dehydration and removal of volatiles around 100 °C, followed by polysaccharide backbone decomposition between 215–290 °C, with complete degradation occurring between 450–550 °C. DTG curves confirm maximum degradation rates (T_max_) between 250–350 °C. All formulations exhibited comparable thermal profiles, indicating that EA incorporation did not significantly alter hydrogel thermal stability.

The thermal stability of KC–EA hydrogels was assessed by thermogravimetric analysis (TGA) and derivative thermogravimetry (DTG) over the temperature range 30–800 °C at a heating rate of 10 °C/min (Fig. 8). The TGA curves represent weight loss as a function of temperature, while the DTG curves indicate the temperature (T_max_) corresponding to the maximum rate of degradation^53^.

All hydrogel formulations exhibited three key thermal degradation events. The initial weight loss near 100 °C was attributed to dehydration and removal of physically adsorbed and bound water, as well as the denaturation of EA proteins and the decomposition of minor volatile compounds^54^. The second major degradation phase, occurring between 215 °C and 290 °C, corresponded primarily to the breakdown of the κ-carrageenan polysaccharide backbone, including cleavage of glycosidic linkages and desulfation of sulfate ester groups, as confirmed by DTG peaks in the 250–350 °C range^55^. The final degradation stage, observed between 450 °C and 550 °C, represented the decomposition of residual protein structures and the complete collapse of the hydrogel matrix.

Importantly, increasing EA content did not markedly shift the degradation pattern, suggesting that thermal stability is predominantly dictated by the κ-carrageenan component, which remained constant across formulations. Nevertheless, EA incorporation contributed to the initial weight loss event by introducing additional protein fractions susceptible to early denaturation. Overall, these findings demonstrate that KC–EA hydrogels maintain substantial thermal resilience, with stability largely governed by carrageenan while EA influences early-stage thermal transitions.

### Mechanical properties of KC–EA hydrogels

#### Strength analysis

**Fig. 9:**
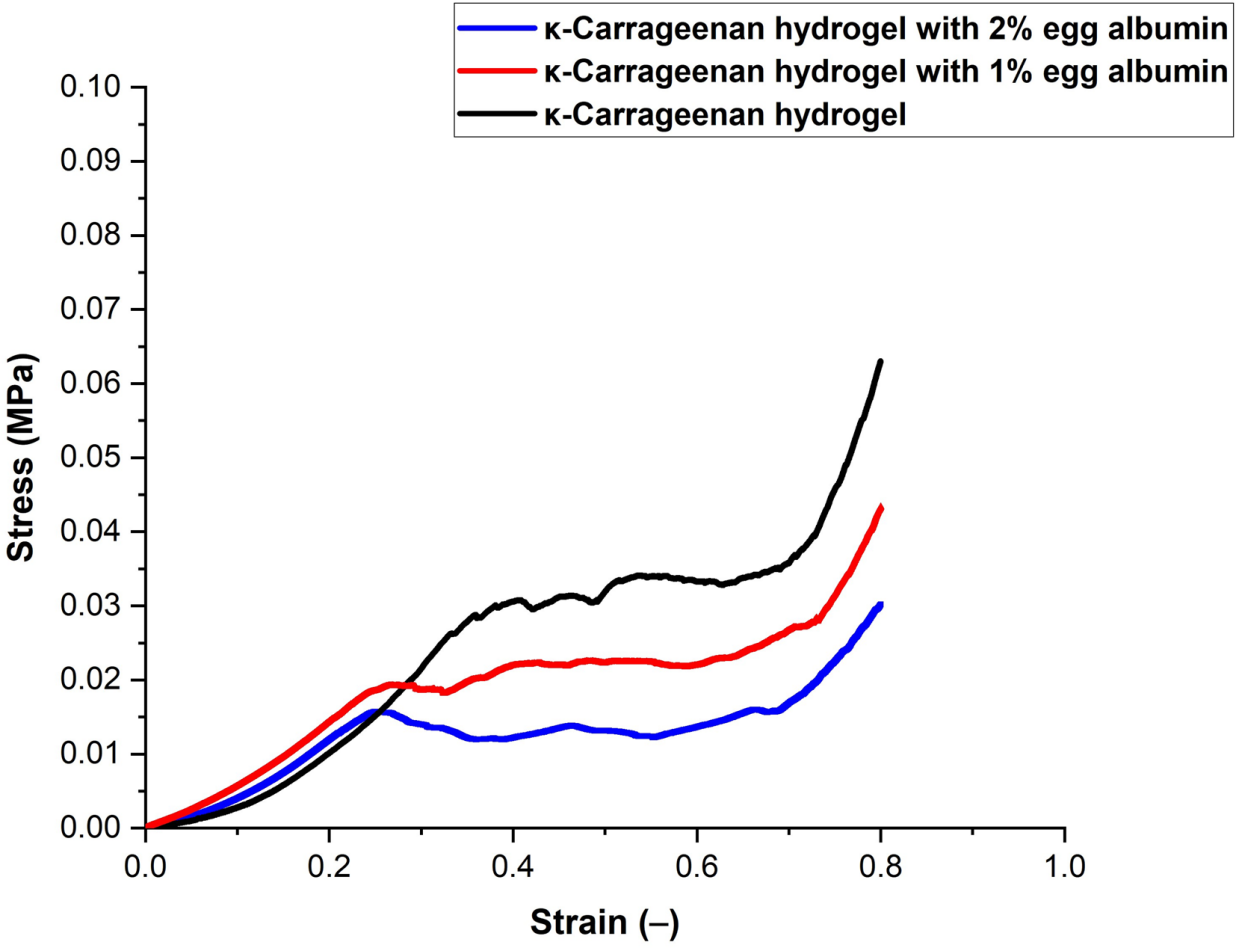
Stress–strain curves of κ-carrageenan (KC) and KC–egg albumin (EA) hydrogels. Pure KC hydrogel exhibited the highest stress values at a given strain, indicating superior stiffness and mechanical strength. Incorporation of EA reduced stiffness and strength, with the 1% EA hydrogel showing moderate softening and the 2% EA hydrogel exhibiting the lowest mechanical strength. These results highlight the concentration-dependent influence of EA on hydrogel flexibility and toughness. Note: Strain (–) is dimensionless and has no units.

The mechanical properties of KC–EA hydrogels were evaluated from stress–strain curves (Fig. 9), which were used to determine Young’s modulus, compressive strength, and energy dissipation (Table 2). Pure KC hydrogels exhibited the highest stress values at a given strain, indicating superior stiffness, strength, and elasticity. The stress increased progressively with strain and culminated in a sharp rise before failure, confirming good structural integrity.

Incorporation of EA reduced the stiffness and strength of KC hydrogels, resulting in softer and more flexible networks. Hydrogels containing 1% EA showed slightly lower stress values than pure KC, reflecting moderate softening due to protein–polysaccharide interactions. At 2% EA, the stress values decreased further, indicating weaker molecular interactions and reduced crosslinking density. This reduction is likely caused by the introduction of additional hydrophilic groups from EA, which increase water uptake and disrupt the compact carrageenan–carragenan ionic junctions. Furthermore, partial unfolding of EA proteins at higher concentrations may interfere with carrageenan helices, loosening the overall network architecture and further reducing effective crosslink density^56^. This trend was consistent with swelling and SEM results, which demonstrated increased water retention and porosity at higher EA concentrations.

Quantitative analysis supported these observations. The KC hydrogel exhibited the highest modulus (0.0769 ± 0.005 MPa), compressive strength (0.0355 ± 0.005 MPa), and energy dissipation (292.521 ± 0.009 J), confirming its superior stiffness and toughness. EA incorporation led to progressive reductions in all parameters, with the 2% EA hydrogel exhibiting the lowest values. Preliminary trials with EA concentrations above 2% produced extremely soft and fragile gels that were unsuitable for handling and testing, while carrageenan concentrations above 3% caused premature gelation during preparation. These findings suggest that further increasing EA content would likely continue to weaken the hydrogel network, yielding mechanically unstable scaffolds.

Overall, EA incorporation produced softer, more flexible hydrogels while reducing mechanical strength. This balance between stiffness and flexibility may be advantageous for biomedical applications requiring adaptable mechanical properties, though considerations of biodegradability and biocompatibility remain essential for clinical translation.

**Table 2:** Mechanical properties of κ-carrageenan (KC) and KC–egg albumin (EA) hydrogels. Pure KC hydrogel exhibited the highest young’s modulus, compressive strength, and energy dissipation, confirming its superior stiffness and toughness. Incorporation of EA reduced these parameters in a concentration-dependent manner, with the 2% EA hydrogel showing the lowest values, consistent with increased softness and flexibility.

Hydrogel	Young’s Modulus (MPa)	Energy (J)	Compressive Strength (MPa)
**κ-Carrageenan**	0.0868 ± 0.003	280.438 ± 0.004	0.0303 ± 0.003
**κ-Carrageenan hydrogel with 1% egg albumin**	0.0747 ± 0.003	217.769 ± 0.007	0.0192 ± 0.007
**κ-Carrageenan hydrogel with 2% egg albumin**	0.0669 ± 0.005	157.521 ± 0.009	0.0155 ± 0.005

#### Physiological degradation

Hydrogel degradation is governed by functional groups such as esters, amides, and imides, which undergo chemical or enzymatic cleavage of polymeric bonds. These processes can manifest as surface erosion, molecular weight reduction, or changes in mechanical performance, reflecting the interconnected nature of physicochemical degradation pathways^57^.

**Fig. 10:**
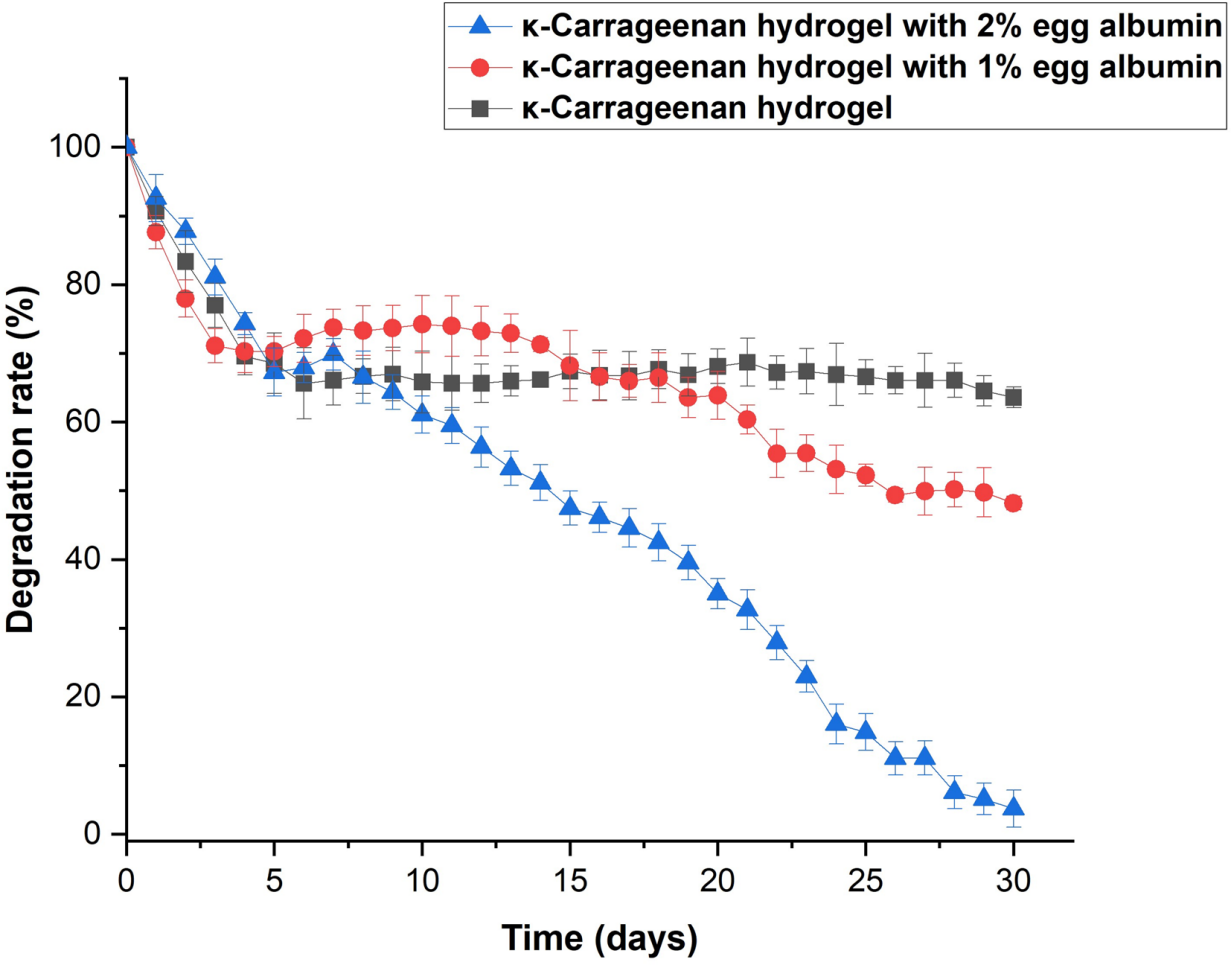
Physiological degradation of κ-carrageenan (KC) and KC–egg albumin (EA) hydrogels in PBS (pH 7.4) at 37 °C over 30 days. Pure KC hydrogels exhibited a relatively stable degradation profile, reflecting the structural integrity of glycosidic linkages. Incorporation of EA accelerated degradation in a concentration-dependent manner, with the 2% EA hydrogel degrading fastest due to enhanced swelling and exposure of hydrophilic functional groups. Data are presented as mean ± SD (*n* = 3).

The degradation behavior of KC–EA hydrogels was assessed at 37 °C in PBS (pH 7.4) (Fig. 10). Pure KC hydrogels exhibited a stable degradation profile, consistent with the structural integrity provided by glycosidic linkages^58^. Incorporation of EA altered this behavior by increasing swelling capacity, attributed to the exposure of hydrophilic functional groups. The enhanced water uptake promoted faster degradation, demonstrating that protein incorporation not only modulates hydrogel hydration but also influences its overall degradation dynamics.

The biodegradation trend followed the order: 2% EA hydrogel > 1% EA hydrogel > pure KC hydrogel, indicating that higher EA content accelerated degradation. This outcome reflects the direct correlation between swelling and degradation, as increased water uptake promotes faster structural breakdown^59^. Mechanical analysis confirmed that higher EA concentrations produced softer scaffolds, which were more prone to degradation under in vitro conditions. FTIR analysis further revealed hydrogen bonding and ionic interactions between KC and EA; however, these bonds are readily disrupted by temperature, pH, and enzymatic activity, facilitating hydrogel fragmentation. Such controlled degradation is advantageous for biomedical applications, as it enhances cell adhesion, migration, and proliferation by providing a dynamic scaffold microenvironment. Therefore, regulating biodegradation rates through EA incorporation represents a key strategy in hydrogel design, ensuring both functionality and biocompatibility within biological systems.

### Biological properties of KC–EA hydrogels

#### Biocompatibility assessment

An ideal scaffold should maintain cell viability while promoting adhesion and proliferation. KC hydrogels are biocompatible but exhibit limited metabolic activity of fibroblasts, largely due to inadequate pore size, poor surface properties, and insufficient ECM mimicry^60^. To overcome these limitations, EA was incorporated into KC hydrogels in the present study, as it is biocompatible and contains cell-recognition sequences that enhance biological interactions^61^.

**Fig. 11:**
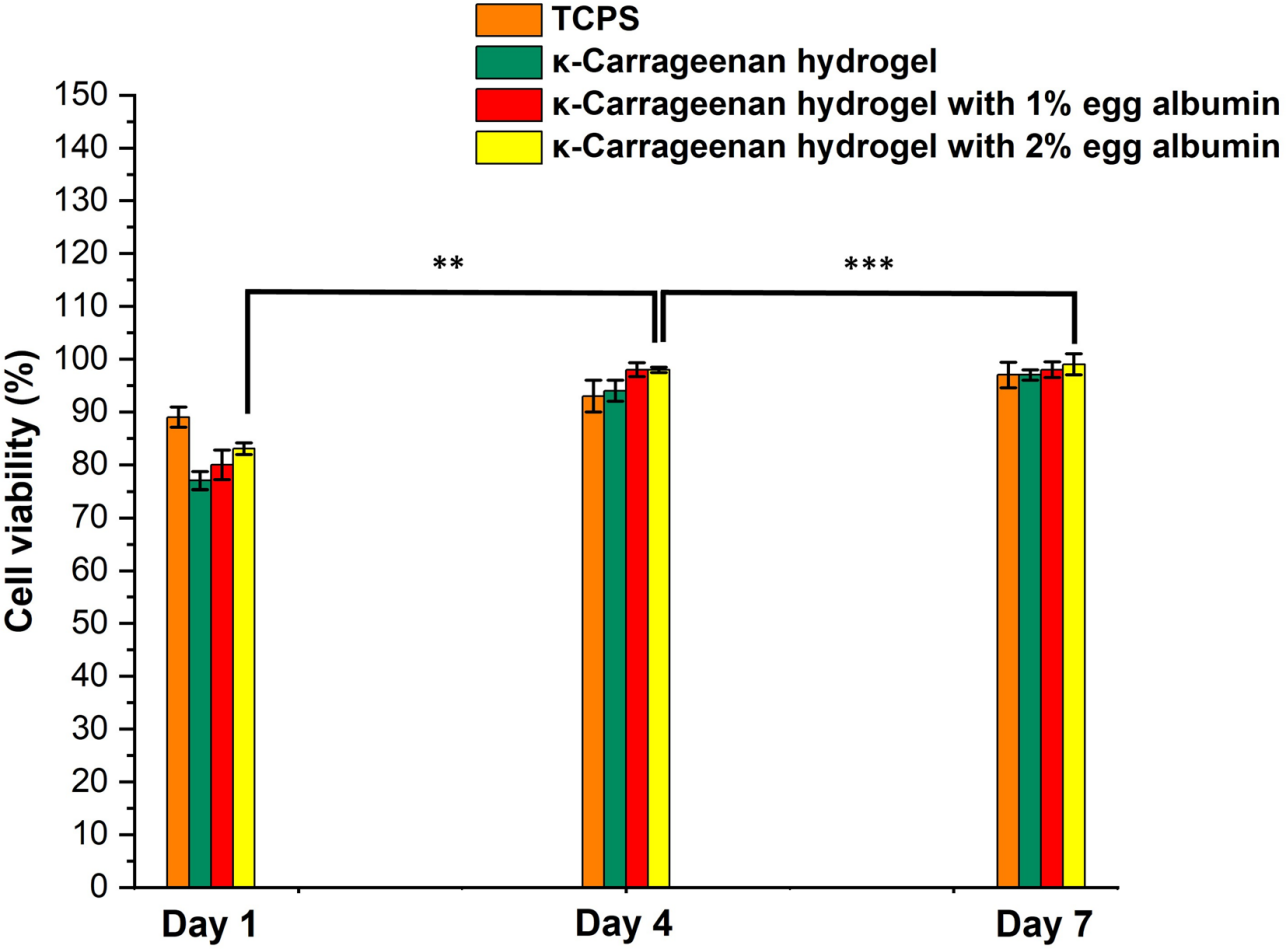
Cell viability of 3T3 fibroblasts cultured on tissue culture polystyrene (TCPS), pure κ-carrageenan hydrogels, and κ-carrageenan hydrogels containing 1% or 2% egg albumin over 1, 4, and 7 days, as determined by the MTT assay. EA incorporation significantly enhanced cell proliferation compared with KC-only hydrogels (**p < 0.01; ***p < 0.001). Data are presented as mean ± SD (*n* = 3).

Biocompatibility was assessed by seeding 3T3 cells onto the hydrogel surfaces and measuring cell viability at days 1, 4, and 7 using the MTT assay (Fig. 11). At all time points, cell proliferation on KC–EA hydrogels was significantly higher than that on KC-only hydrogels. This improvement is attributed to the Arg-Tyr-Asp-Ser (RYDS) sequence in EA, which mimics the Arg-Gly-Asp-Ser (RGDS) motif known for mediating integrin-dependent cell attachment^62–64^. KC also contributes by mimicking natural glycosaminoglycans, providing a biomimetic environment that supports cellular interactions^65^. Furthermore, the physicochemical properties of the hydrogels, resembling the hydrated state of native ECM, allowed efficient nutrient and oxygen transport. Among the tested formulations, the hydrogel with 2% EA exhibited the highest cell viability, likely due to increased surface heterogeneity (Fig. 7*(c)*) and exposure of hydrophilic groups, which promoted cell adhesion and proliferation^63^.

**Fig. 12:**
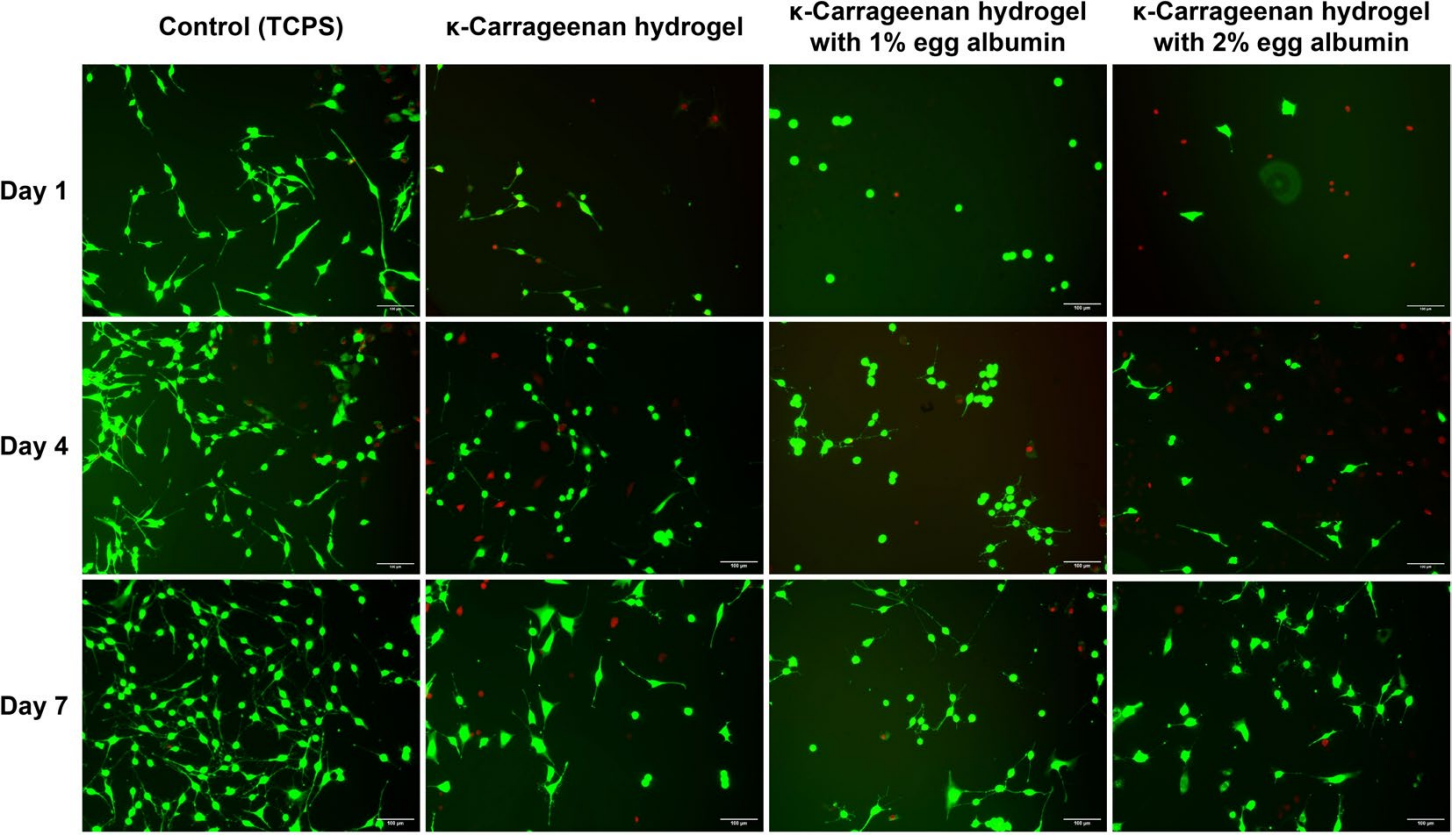
Live/dead fluorescence images of 3T3 fibroblasts cultured on tissue culture polystyrene (TCPS, control), pure κ-carrageenan hydrogels, and κ-carrageenan hydrogels containing 1% or 2% egg albumin at days 1, 4, and 7. Live cells were stained with calcein-AM (green), while dead cells were stained with ethidium homodimer-1 (red). EA incorporation enhanced cell adhesion and proliferation compared with pure KC hydrogels, with the 2% EA hydrogel supporting the highest cell viability by day 7. Scale bars = 100 μm.

Cell morphology was further examined by live/dead staining and fluorescence microscopy (Fig. 12). On day 1, only a few fusiform-shaped cells with incomplete spreading were observed, consistent with the initial adaptation phase of cells to the hydrogel surface. By day 4, cells displayed more elongated, spindle-like morphology with enhanced spreading and intercellular connections, indicative of cytoskeletal organization and active proliferation. By day 7, cells exhibited dense fibroblast-like morphology, extensive spreading, and confluent monolayer formation, with evidence of mature cytoskeletal structures and possible ECM deposition. This temporal progression indicates that EA incorporation not only facilitated early adhesion but also sustained long-term cell proliferation and matrix remodeling. These findings are consistent with prior reports that EA enhances scaffold biocompatibility by promoting integrin-mediated adhesion and cell spreading^47,66^.

Collectively, these results demonstrate that EA incorporation enhances the biocompatibility of KC hydrogels by providing biochemical cues, improving surface morphology, and increasing hydrophilicity. These properties create a more favorable cellular microenvironment, underscoring the potential of KC–EA hydrogels as tissue engineering scaffolds.

## Conclusion

Egg albumin remains one of the least explored biopolymers despite its abundance, cost-effectiveness, and diverse biochemical properties. In this study, EA was extracted from hen eggs and incorporated into κ-carrageenan hydrogels to evaluate its potential as a copolymer for biomedical applications.

Incorporation of EA significantly altered the physicochemical and mechanical behavior of KC hydrogels. EA increased hydrophilicity, leading to higher water absorption and accelerated degradation through bond cleavage within the polymer network. FTIR analysis confirmed electrostatic interactions between the negatively charged sulfate ester groups of KC and the positively charged domains of EA, supporting protein–polysaccharide compatibility. SEM revealed that EA addition smoothed the hydrogel surface but introduced irregular aggregates, while swelling and degradation studies demonstrated that higher EA concentrations enhanced water uptake and promoted faster structural breakdown.

Mechanical testing showed that pure KC hydrogels exhibited superior stiffness and strength, whereas EA incorporation reduced mechanical integrity, resulting in softer and more flexible scaffolds. This reduction is likely due to disruption of the compact KC network and weaker intermolecular interactions. Nevertheless, the increased softness and structural heterogeneity may contribute to enhanced toughness and flexibility—properties favorable in certain biomedical contexts. Most importantly, EA incorporation markedly improved biocompatibility. EA-enriched hydrogels supported greater cell adhesion, proliferation, and viability compared with pure KC, attributable to protein-derived recognition sequences and enhanced hydrophilicity.

Collectively, these findings demonstrate that EA incorporation provides a strategy to tune the physicochemical, mechanical, and biological properties of KC hydrogels. By balancing mechanical performance with improved degradation and cytocompatibility, KC–EA hydrogels hold promise as adaptable biomaterials for soft tissue engineering applications. Future research should focus on integrating therapeutic agents or growth factors to extend their applicability to advanced tissue engineering and wound healing systems.

## Data Availability

All the data that supports the findings of this study are included in this published article.
